# Supplementary material for: A biodegradable and flexible neural interface for transdermal optoelectronic modulation and regeneration of peripheral nerves
Source: Nat Commun. 2024 Jun 3;15:4721. doi: 10.1038/s41467-024-49166-4 (PMC11148186; doi:10.1038/s41467-024-49166-4)
Supplement: Supplementary file 1 — Supplementary information [file 41467_2024_49166_MOESM1_ESM.pdf]

Supplementary information

A biodegradable and flexible neural interface for transdermal optoelectronic modulation and regeneration of peripheral nerves

Pengcheng Sun, Chaochao Li, Can Yang, Mengchun Sun, Hanqing Hou, Yanjun Guan, Jinger Chen, Shangbin Liu, Kuntao Chen, Yuan Ma, Yunxiang Huang, Xiangling Li, Huachun Wang, Liu Wang, Shengfeng Chen, Haofeng Cheng, Wei Xiong, Xing Sheng, Milin Zhang, Jiang Peng, Shirong Wang*, Yu Wang*, Lan Yin*

This PDF file includes:

Supplementary Figures 1 to 16

Supplementary Figure 1

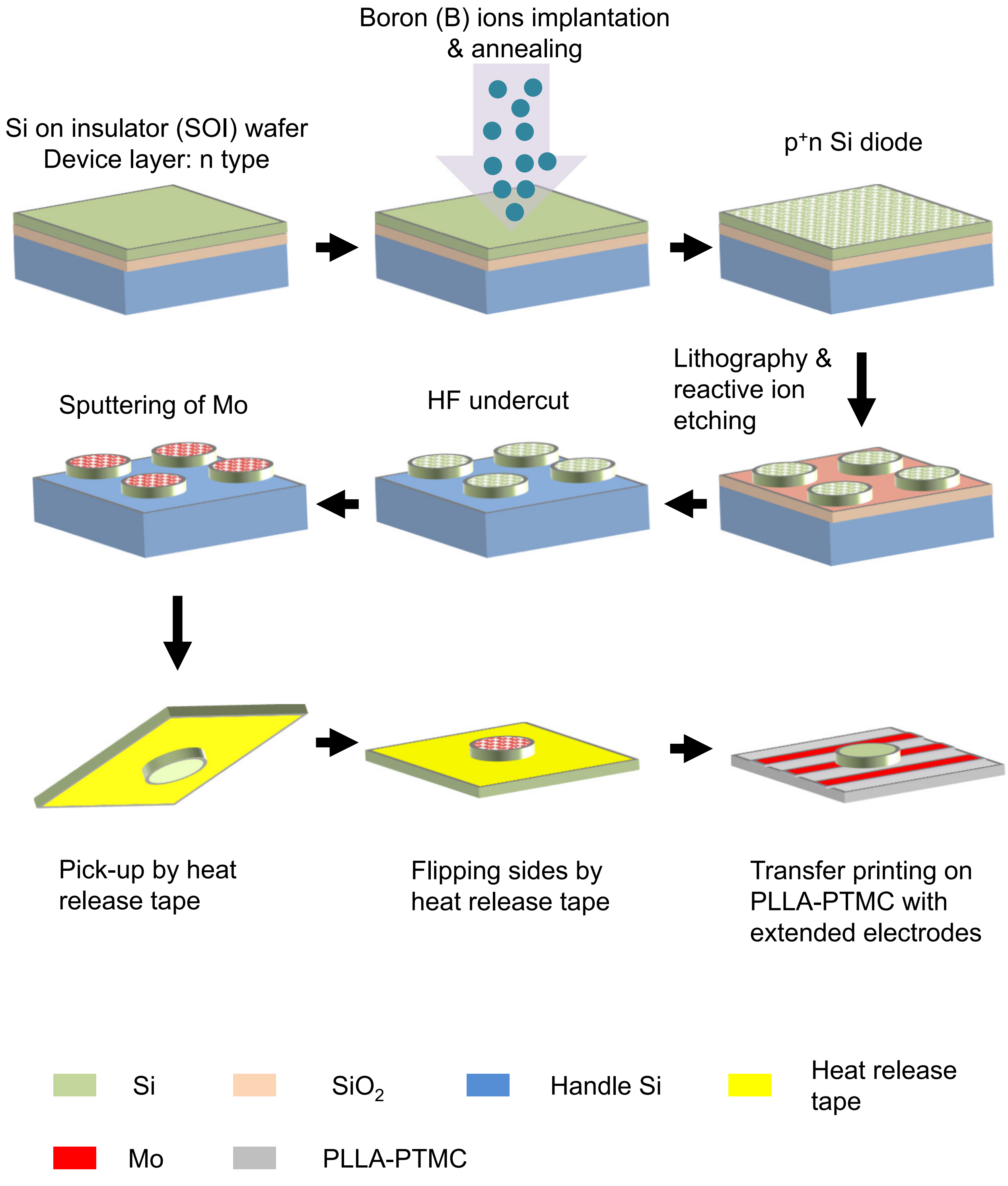

Supplementary Fig. 1 | Fabrication process of the biodegradable and flexible optoelectronic neural interface.

Supplementary Figure 2

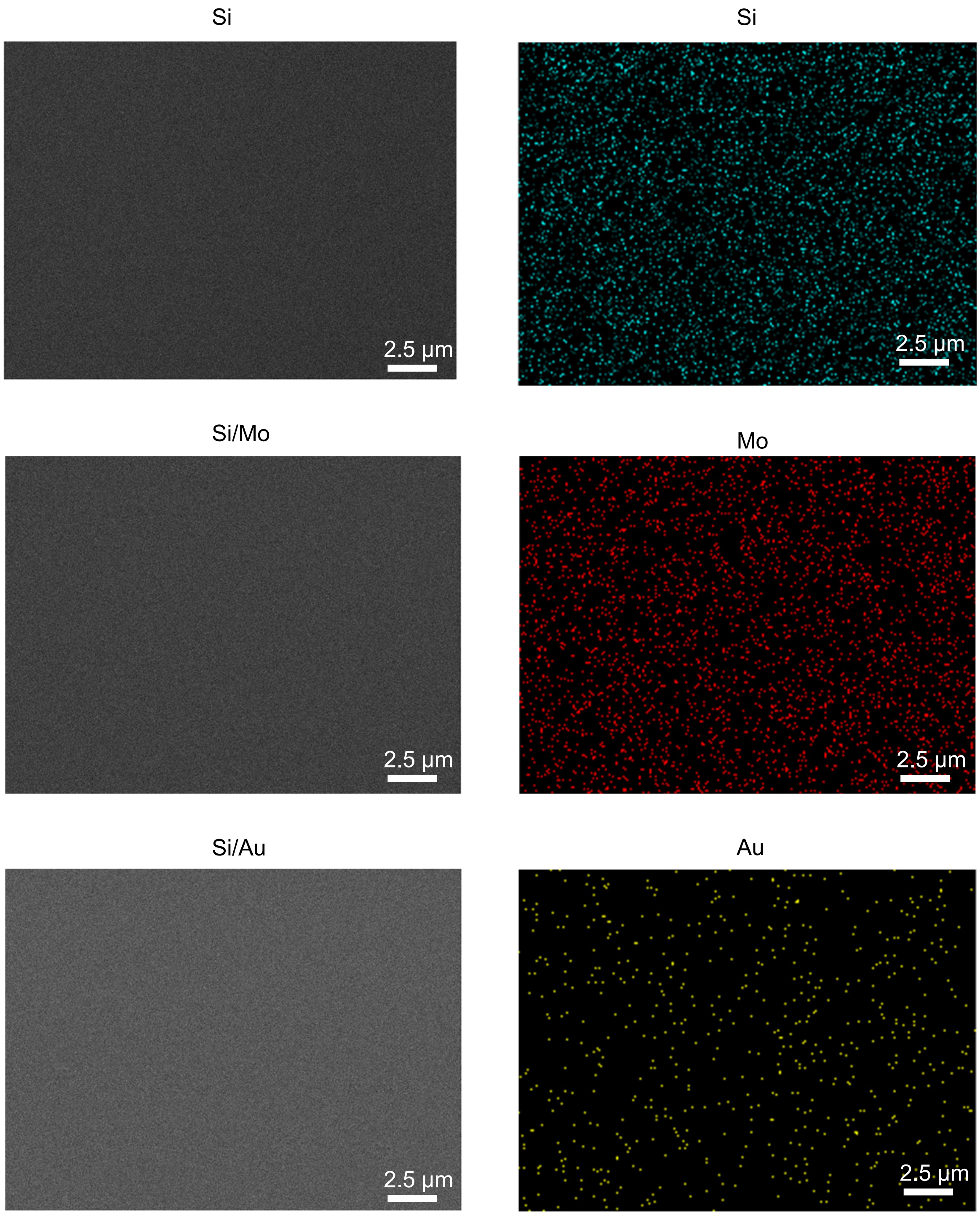

Supplementary Fig. 2 | Scanning electron microscope (SEM) and energy-dispersive X-ray spectroscopy (EDS) results of the p⁺ surface of the Si diodes without a modification layer (Si devices), Si diodes with Mo modification layer (Si/Mo devices) and Si diode with Au modification layer (Si/Au devices). ***n*** **= 3 samples.**

Supplementary Figure 3

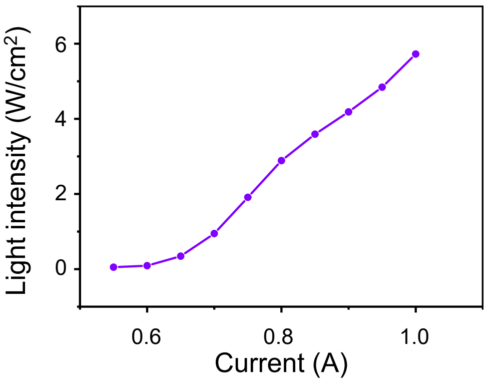

Supplementary Fig. 3 | Current reading of 635 nm laser diode and its corresponding output light intensity.

Supplementary Figure 4

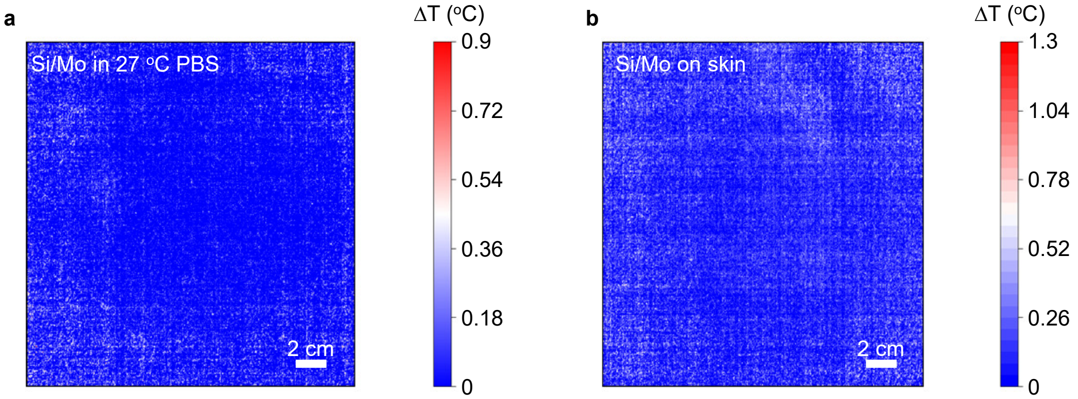

Supplementary Fig. 4 | Temperature changes upon laser illumination on the Si-based optoelectronic device, captured by an infrared thermal imaging camera. a Temperature changes of the Si/Mo device surface after 5 s of pulse laser irradiation (635 nm, 10 Hz, pulse width 10 ms, 0.95 W/cm^2) in PBS solution (27 °C, pH 7.4). **b** Temperature changes of the Si/Mo device placed on the facial skin of New Zealand rabbits after 1 hour of pulse laser irradiation (635 nm, 10 Hz, pulse width 10 ms, 0.95 W/cm^2). Laser is incident from the back side and passes through the skin. In **a**, **b**, *n* = 3 samples.

Supplementary Figure 5

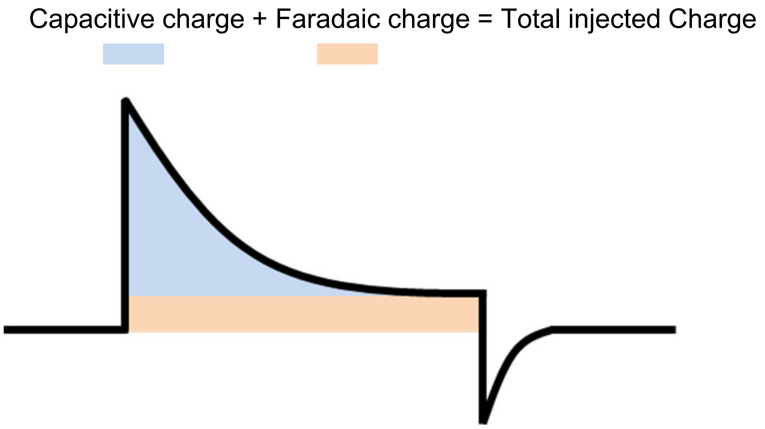

Supplementary Fig. 5 | Schematic diagram of a photocurrent transient (black line) under laser illumination, in which the capacitive charge, faradaic charge, and total injected charge can be defined.

Supplementary Figure 6

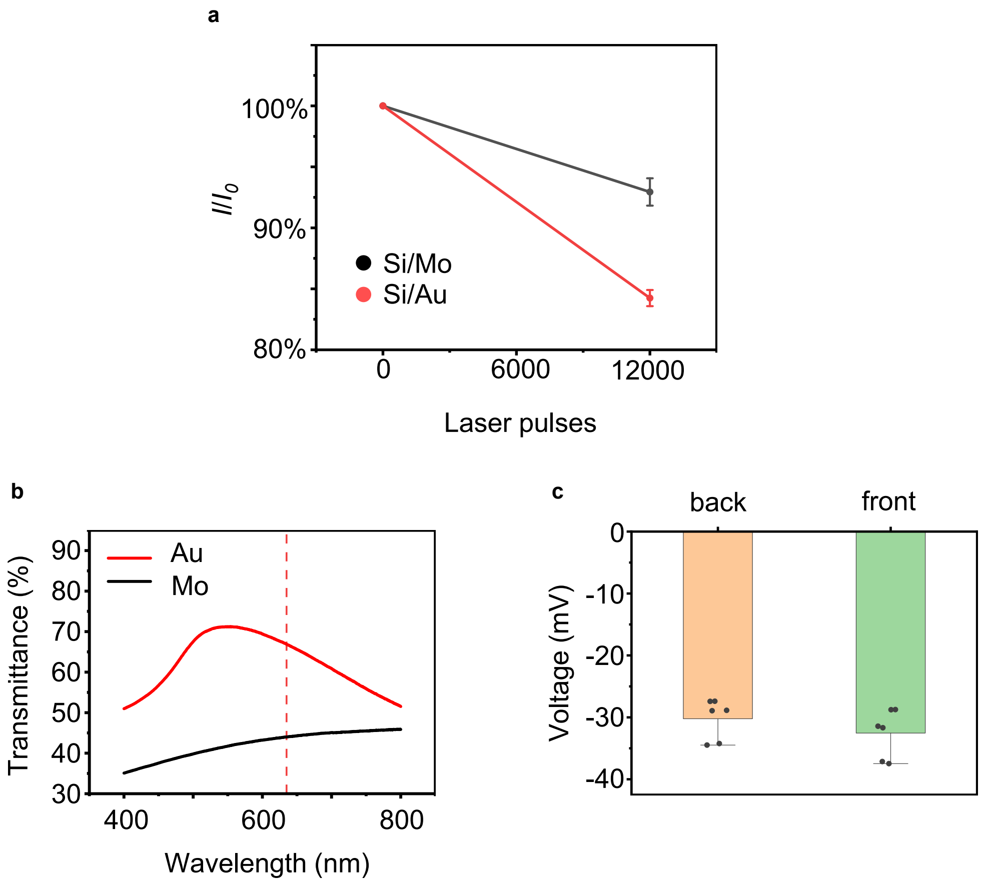

Supplementary Fig. 6 | a Photocurrent shows high retention after 12000 pulse cycles (635 nm, 0.95 W/cm^2, 10 Hz, pulse width 10 ms). I: measured photocurrent; I_0: initial photocurrent. **b** Light transmission through Au (~10 nm) or Mo (~10 nm) modification layers. **c** Photoresponses under illumination (635 nm, 10 Hz, pulse width 10 ms, 0.95 W/cm^2) incident from the front side and back side (transmitted through the modification layer and separated extended electrodes) of Si/Mo devices. In **a**, n = 3 samples in each group. In **c**, n = 6 samples in each group. All data are presented as mean ± s.e.m.

Supplementary Figure 7

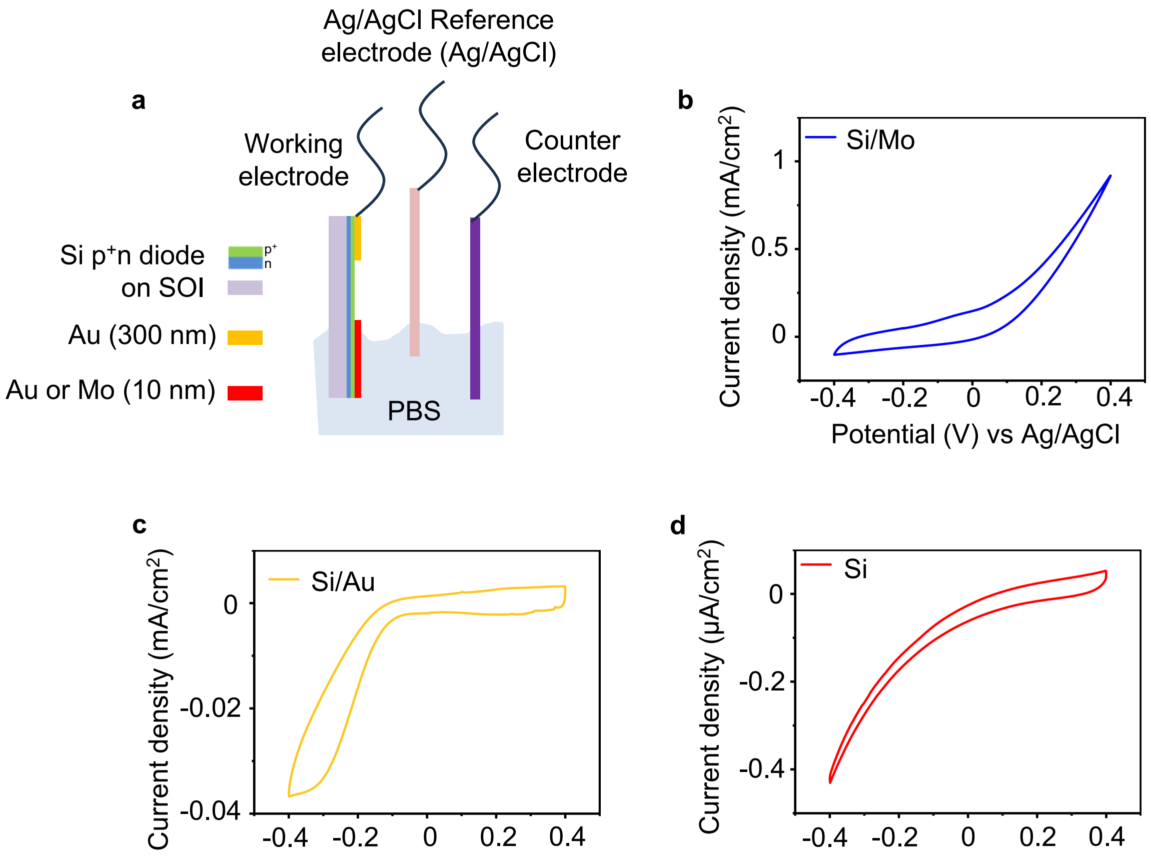

Supplementary Fig. 7 | a Schematic diagram of the electrochemical measurement. **b, c** and **d** Representative CV curves of Si/Mo devices, Si/Au devices and Si devices (no modification layer). In **b**–**d**, *n* = 3 samples.

Supplementary Figure 8

a

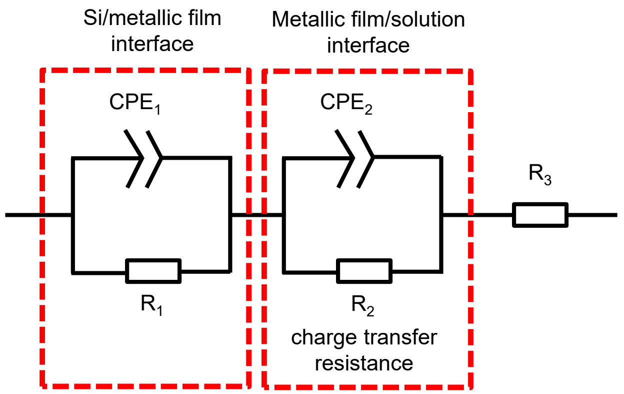

b

	R_1 (Ω)	R_2 (Ω)	R_3 (Ω)	$CPE\text{-}T_1$ ($s^n/Ω$)	CPE-P_1	CPE-T_2 ($s^n/Ω$)	CPE-P_2	C_1(F)	C_2(F)
Si/Mo	154.4	1480	163	1.38×10^{-6}	0.705	8.49×10^{-4}	0.744	4.47×10^{-8}	9.18×10^{-4}
Si/Au	92	19604	83.3	3.02×10^{-7}	0.816	8.13×10^{-6}	0.879	2.27×10^{-8}	6.31×10^{-6}

c

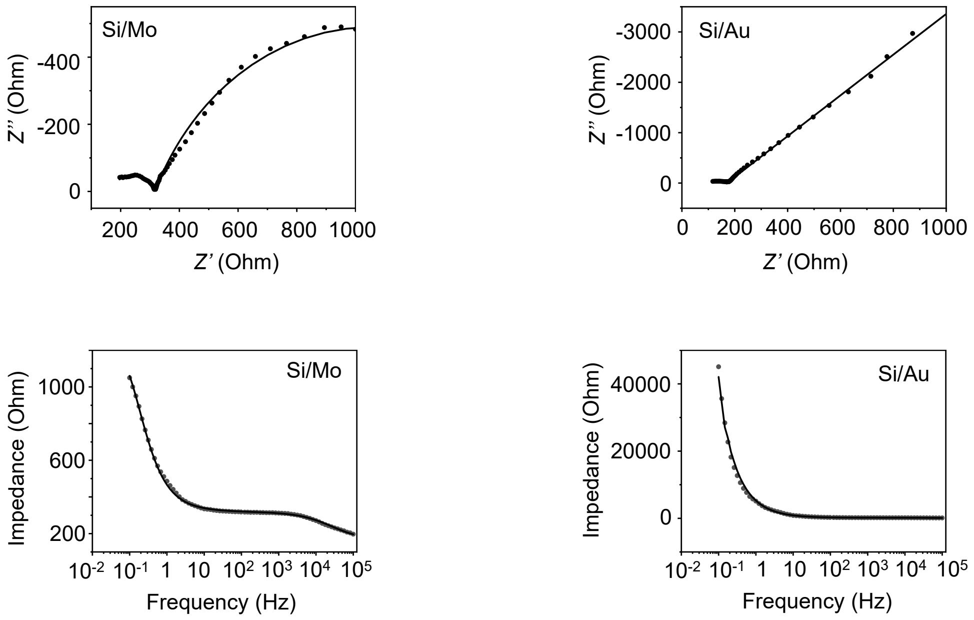

Supplementary Fig. 8 | Equivalent circuit models of electrochemical impedance. a Equivalent circuit of the electrochemical impedance measurement. The parallel circuit of R_1 and CPE_1 (constant phase element) is equivalent to the interface between Si and the metallic modification film. The parallel circuit of R_2 (charge transfer resistance) and CPE_2 is equivalent to the interface between the metallic modification film and PBS. R_3 is solution resistance. **b** Circuit model fitting parameters. **c** Measured electrochemical impedance and model fitting results (solid lines) of Si/Mo and Si/Au devices. In **c**, n = 3 samples.

Supplementary Figure 9

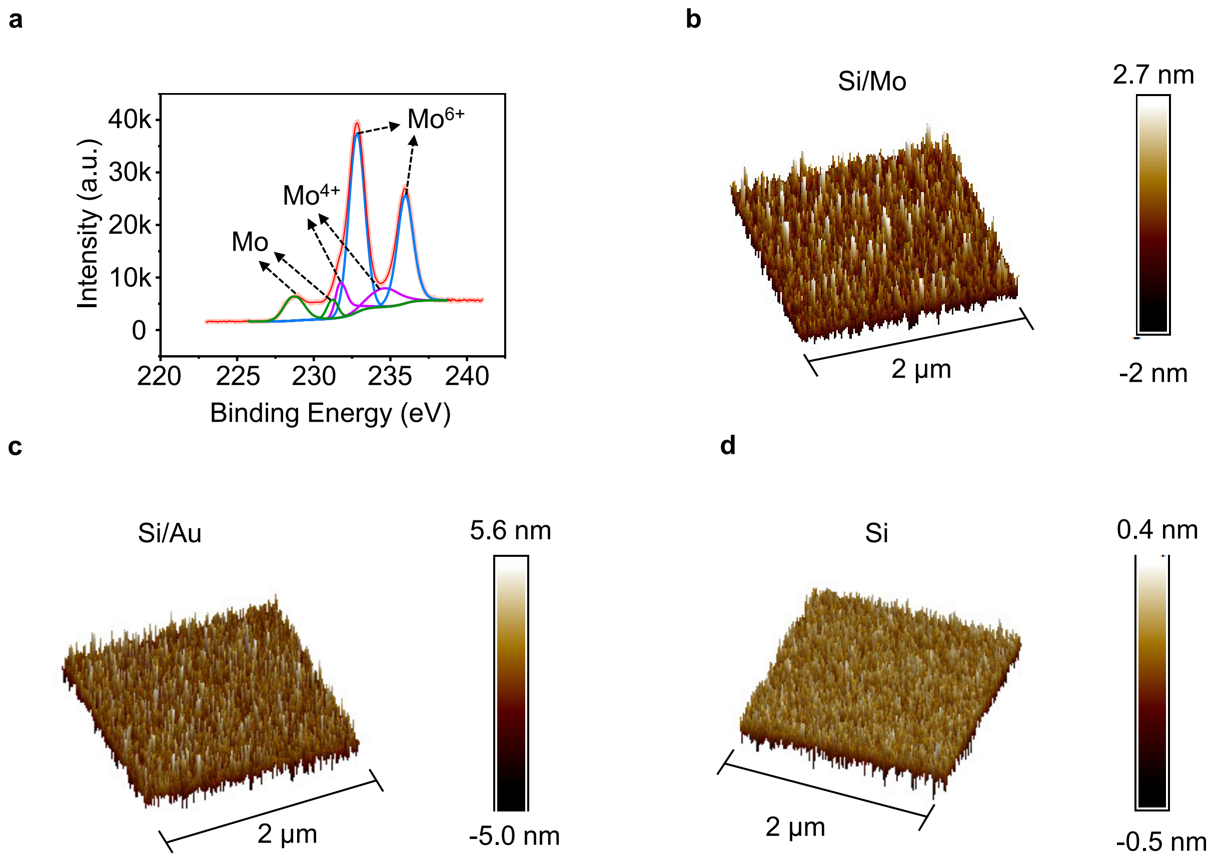

Supplementary Fig. 9 | a Representative X-ray photoelectron spectroscopy (XPS) analysis of the Mo modification layer of Si/Mo devices before CV cycling. **b, c, d** Three-dimensional reconstruction of the p^+ side of the Si/Mo, Si/Au, and Si devices obtained by scanning with an atomic force microscope (AFM). In **a**–**d**, $n = 3$ samples in each group.

Supplementary Figure 10

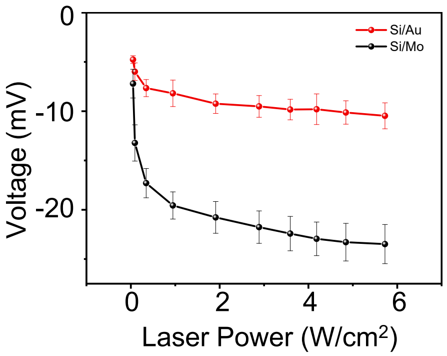

Supplementary Fig. 10 | Measured photovoltage of Si/Mo and Si/Au devices with a diameter of 2 mm at different light intensities (635 nm, 10 Hz, pulse width 10 ms). *n* = 5. All data are presented as mean ± s.e.m.

Supplementary Figure 11

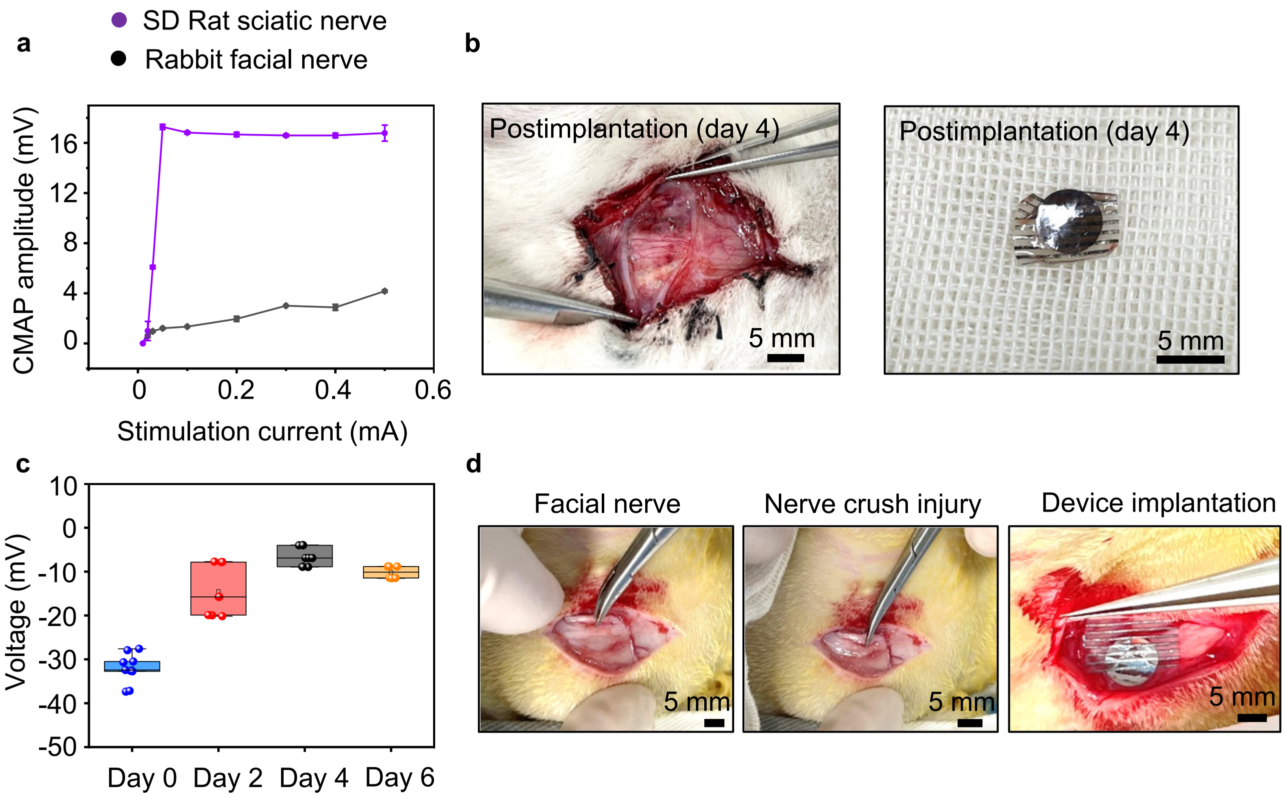

Supplementary Fig. 11 | a CMAP amplitudes of muscles evoked by stimulating the sciatic nerve in rats and the facial nerve in New Zealand rabbits with different currents. **b** Si/Mo devices retrieved from the implantation site after 4 days of implantation. The device remains almost intact. **c** Measured photovoltage of Si/Mo devices before and after implantation over a 6-day time frame (light intensity: 0.95 W/cm^2). **d** Images of the anatomy of the facial nerve, crush injury, and device implantation. In **a**, **c**, n = 3 independent experiments. In **b**, **d**, n = 4 independent experiments. All data are presented as mean ± s.e.m. The box plot presents the median (center line), lower quartile (lower border), upper quartile (upper border), maximum (upper whisker) and minimum (lower whisker), which are ≤ 1.5 times the interquartile range.

Supplementary Figure 12

a

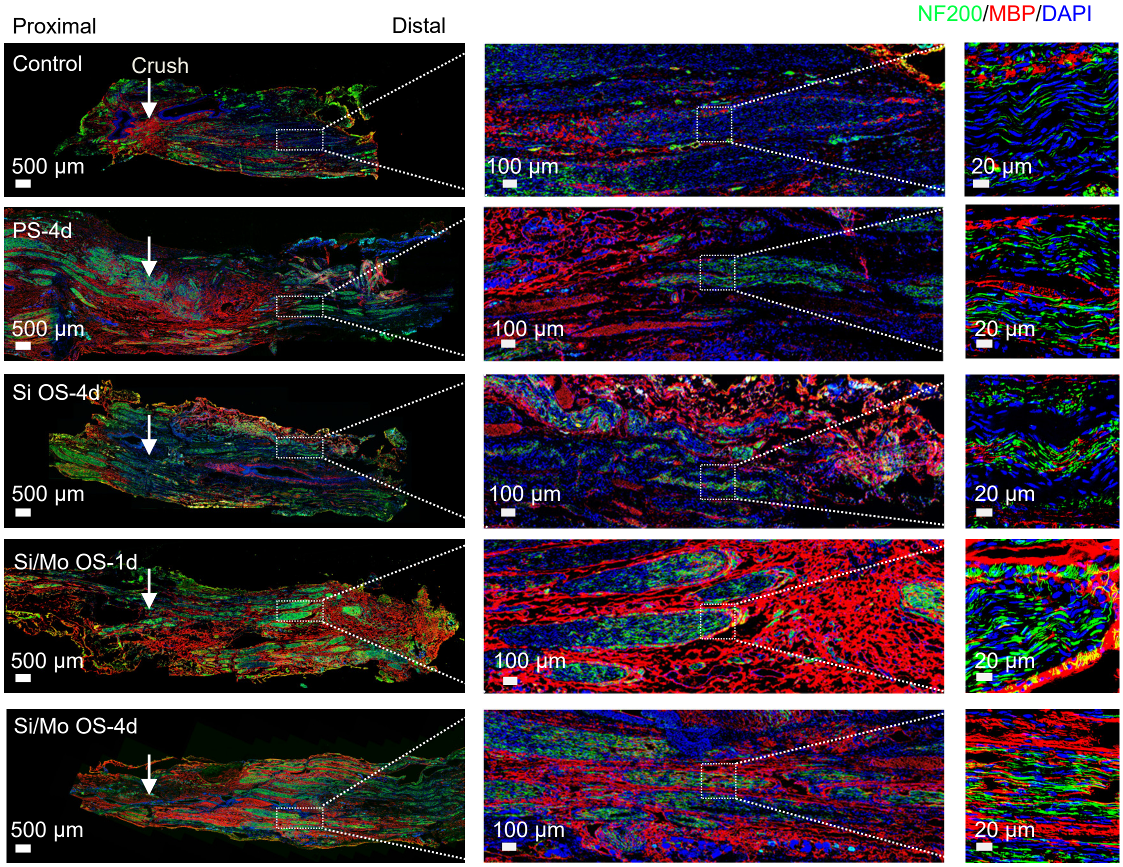

b

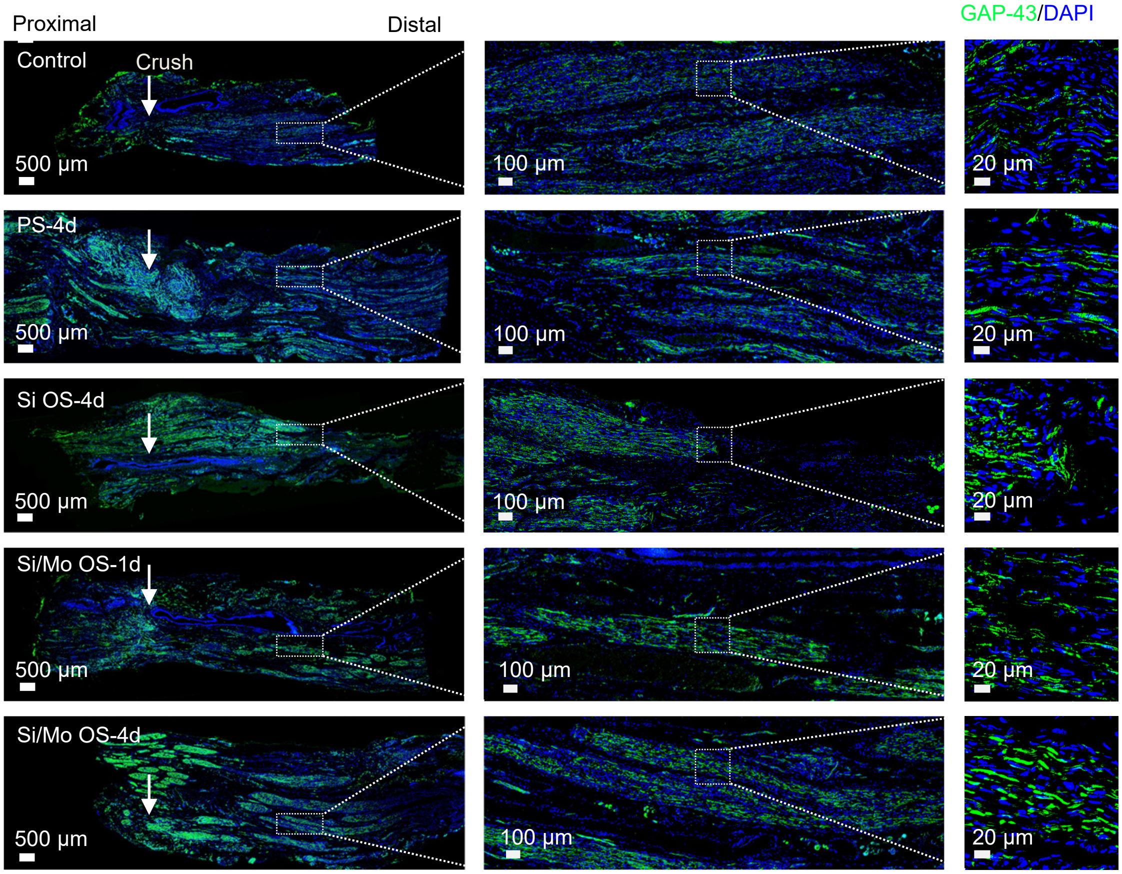

Supplementary Fig. 12 | Immunofluorescence staining images of the longitudinally sectioned regenerated nerve segments at 4 weeks postoperatively. a Immunohistochemical staining: axons (NF200, green), myelin sheaths (MBP, red), and nuclei (DAPI, blue). **b** Immunohistochemical staining: newly grown axons (GAP-43, green), and nuclei (DAPI, blue). White arrow indicates the site of crush injuries. Phototherapy is applied 1-h daily for 1 or 4 days. Groups: Control (no treatment), PS-4d (photostimulation for 4 days), Si OS-4d (optoelectronic stimulation for 4 days with Si devices), Si/Mo OS-1d (optoelectronic stimulation for 1 day with Si/Mo devices), Si/Mo OS-4d (optoelectronic stimulation for 4 days with Si/Mo devices). In **a**, **b**, *n* = 4 independent experiments.

Supplementary Figure 13

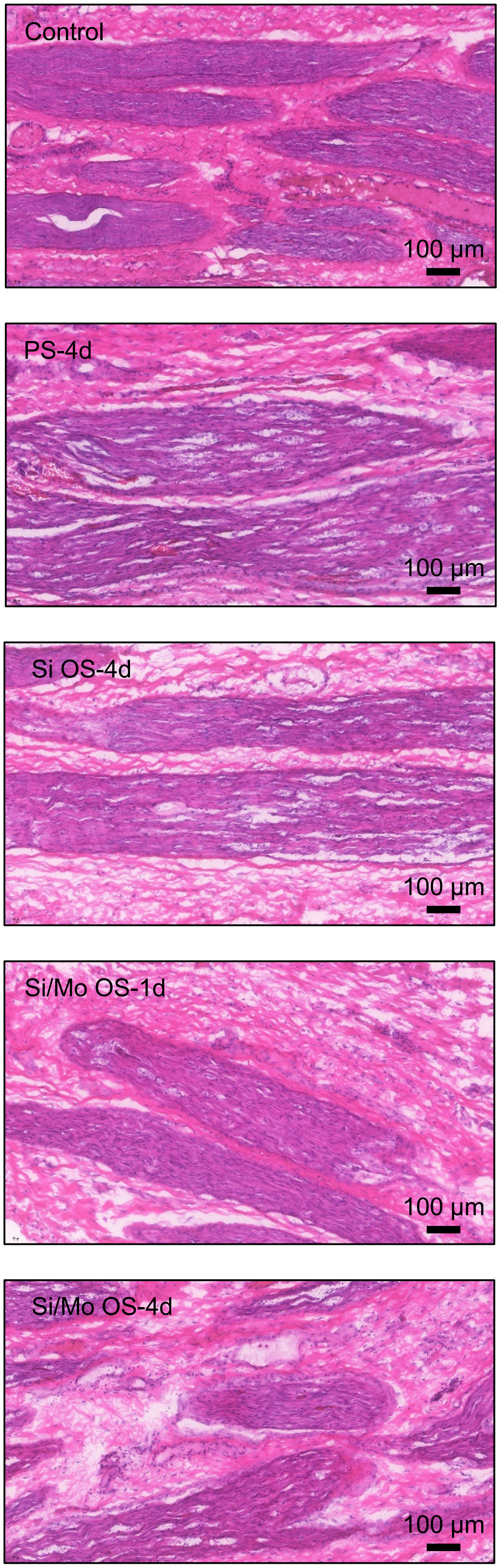

Supplementary Fig. 13 | H&E staining images of the longitudinally sectioned regenerated nerve segments at 4 weeks postoperatively. Phototherapy is applied 1-h daily for 1 or 4 days. Groups: Control (no treatment), PS-4d (photostimulation for 4 days), Si OS-4d (optoelectronic stimulation for 4 days with Si devices), Si/Mo OS-1d (optoelectronic stimulation for 1 day with Si/Mo devices), Si/Mo OS-4d (optoelectronic stimulation for 4 days with Si/Mo devices). *n* = 4 independent experiments.

Supplementary Figure 14

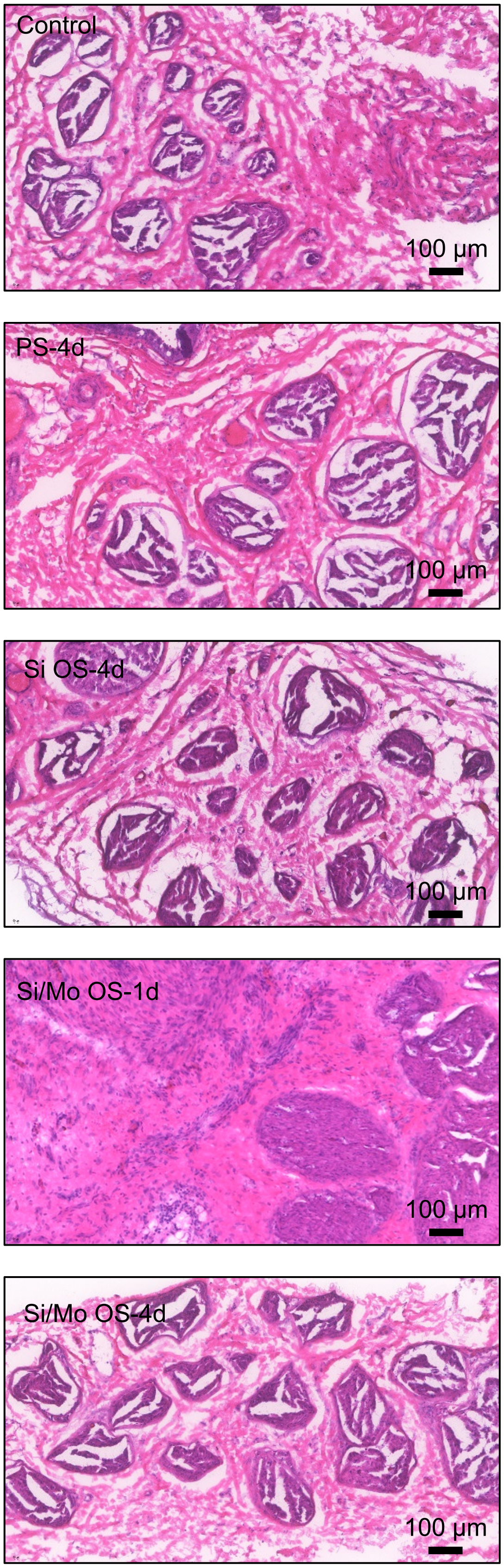

Supplementary Fig. 14 | H&E staining images of the transversely sectioned regenerated nerve segments at 8 weeks postoperatively. Phototherapy is applied 1-h daily for 1 or 4 days. Groups: Control (no treatment), PS-4d (photostimulation for 4 days), Si OS-4d (optoelectronic stimulation for 4 days with Si devices), Si/Mo OS-1d (optoelectronic stimulation for 1 day with Si/Mo devices), Si/Mo OS-4d (optoelectronic stimulation for 4 days with Si/Mo devices). *n* = 4 independent experiments.

Supplementary Figure 15

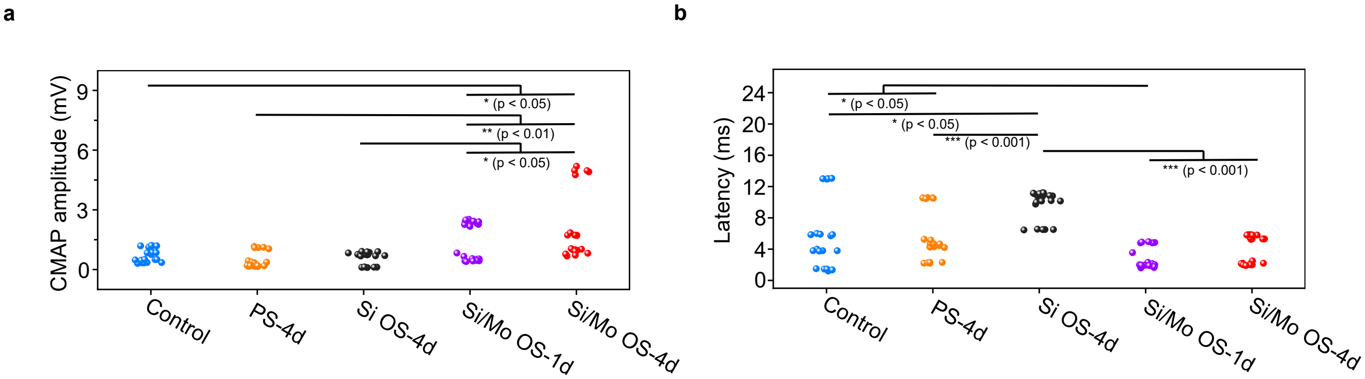

Supplementary Fig. 15 | Statistical analysis of CMAP at 4 weeks postoperatively. a CMAP amplitude at 4 weeks postoperatively. **b** Latency of CMAPs at 4 weeks postoperatively. Phototherapy is applied 1-h daily for 1 or 4 days. Groups: Control (no treatment), PS-4d (photostimulation for 4 days), Si OS-4d (optoelectronic stimulation for 4 days with Si devices), Si/Mo OS-1d (optoelectronic stimulation for 1 day with Si/Mo devices), Si/Mo OS-4d (optoelectronic stimulation for 4 days with Si/Mo devices). In **a**, **b**, $n = 4$ independent experiments. Statistics is analyzed through SPSS (version 23.0), followed by one-way ANOVA (* $p < 0.05$, ** $p < 0.01$, *** $p < 0.001$).

Supplementary Figure 16

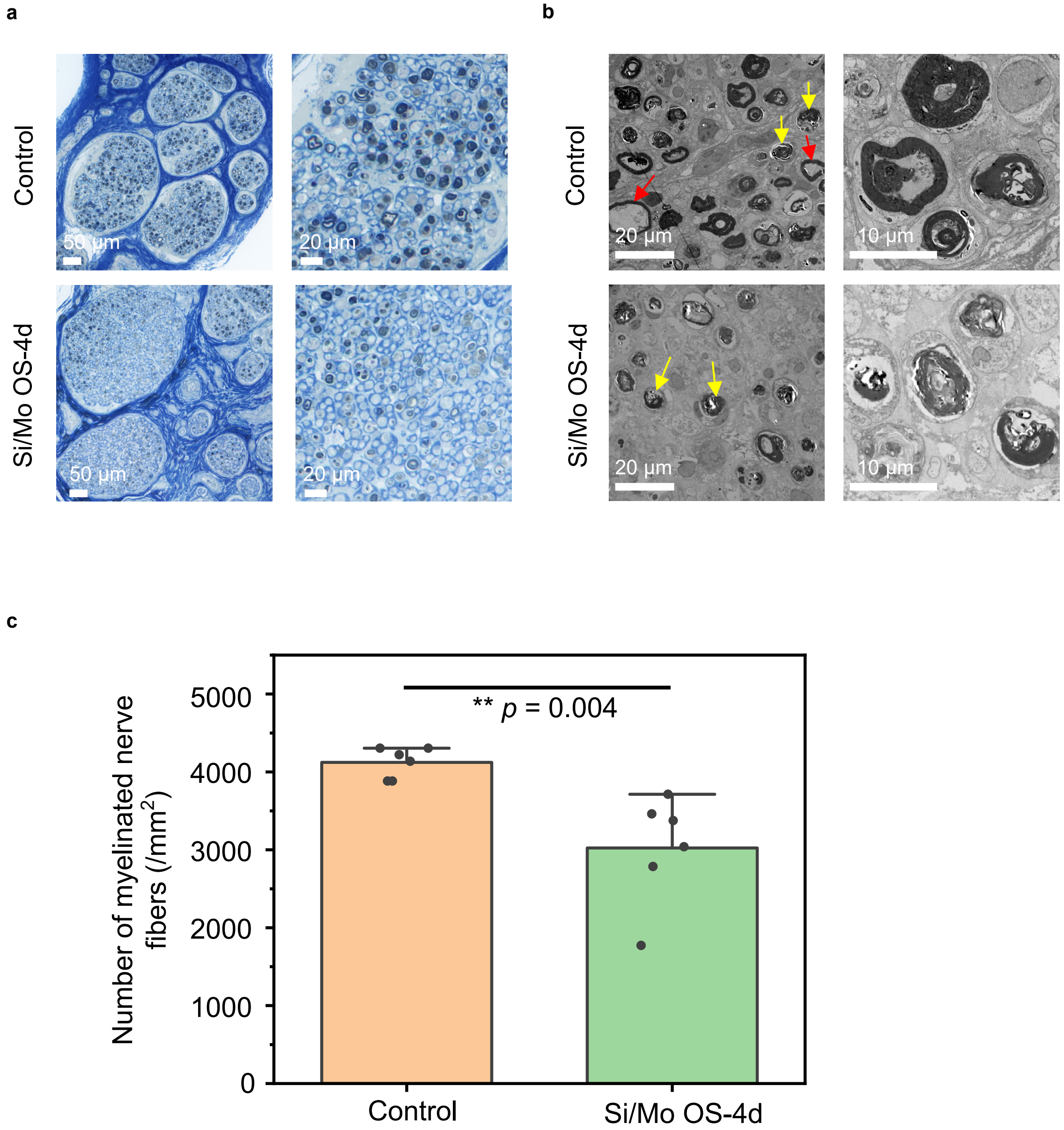

Supplementary Fig. 16 | Effects of optoelectronic stimulation on Wallerian degeneration in facial nerve crush injury. a Toluidine Blue staining of the transversely sectioned nerve segment located 10 mm distal from the crush site on day 7 postoperatively. **b** TEM images of the transversely sectioned nerve segments located 10 mm distal from the crush site on day 7 postoperatively. **c** The density of myelinated nerve fibers based on toluidine blue staining results. Myelinated nerve fibers include both the degenerating (indicated by the yellow arrow) and intact (indicated by the red arrow) nerve fibers on day 7 postoperatively. Groups: Control (no treatment), Si/Mo OS-4d (optoelectronic stimulation for 4 days with Si/Mo devices). In **a**, **b**, **c**, n = 3 independent experiments. All data are presented as mean ± s.e.m. Statistics is analyzed through SPSS (version 23.0), followed by one-way ANOVA (* $p < 0.05$, ** $p < 0.01$, *** $p < 0.001$).
