# Supplementary material for: A biodegradable and flexible neural interface for transdermal optoelectronic modulation and regeneration of peripheral nerves
Source: Nat Commun. 2024 Jun 3;15:4721. doi: 10.1038/s41467-024-49166-4 (PMC11148186; doi:10.1038/s41467-024-49166-4)
Supplement: Supplementary file 3 — Description of Additional Supplementary Files [file 41467_2024_49166_MOESM3_ESM.pdf]

Description of Additional Supplementary Files

Supplementary Movie 1 | The excitation of sciatic nerves and resulted movement of the right hind limb of SD rats under the illumination of a red laser beam (635 nm, 10 Hz, 10 ms pulse width, intensity 0.95 W/cm^2). The Si/Mo device triggers significantly greater movement compared with the Si/Au device.
